# Mood and behavior seasonality in glaucoma; assessing correlations between seasonality and structure and function of the retinal ganglion cells

**DOI:** 10.1371/journal.pone.0229991

**Published:** 2020-03-12

**Authors:** Helle Østergaard Madsen, Shakoor Ba-Ali, Henrik Lund-Andersen, Klaus Martiny, Ida Hageman

**Affiliations:** 1 Mental Health Center Copenhagen, Copenhagen, Denmark; 2 Department of Ophthalmology, Rigshospitalet-Glostrup Hospital, Copenhagen, Denmark; 3 Mental Health Services, Capital Region of Denmark, Copenhagen, Denmark; Universidade Federal do Rio de Janeiro, BRAZIL

## Abstract

**Aim:**

In glaucoma, depression and disturbed sleep has been associated with degeneration of the intrinsically photosensitive retinal ganglion cells, that mediate non-image forming effects of light such as regulation of circadian rhythm, alertness and mood. In this study we assessed associations between seasonal mood and behavior variation and retinal ganglion cell damage in outpatients with glaucoma.

**Methods:**

The seasonal pattern assessment questionnaire was administered to outpatients with glaucoma. Data on visual field defects identified by autoperimetry and retinal nerve fiber layer thickness visualized by ocular coherence tomography were collected from patient charts. The correlations between seasonality and retinal damage were tested and the adjusted effects of retinal function on seasonality were evaluated in a linear regression model.

**Results:**

In total, 113 persons completed the questionnaire. Of these, 4% fulfilled the criteria for seasonal affective disorder (SAD) and 8% for subsyndromal seasonal affective disorder (sSAD). Mean global seasonal score was 4.3. There were no significant correlations between seasonality and either visual field or retinal nerve fiber layer thickness. In the adjusted analysis there were trends toward differential effects of visual field on seasonality in subgroups with different sex and type of glaucoma.

**Conclusion:**

There were no strong associations between seasonality and visual field or retinal nerve fiber layer thickness. Sex, age and glaucoma subtype may modify light effects on complex regulatory systems.

## Introduction

In glaucoma, a neurodegenerative process targets the 1.1 million retinal ganglion cells of the human retina. Approximately one percent of these can be identified as intrinsically photosensitive retinal ganglion cells (ipRGCs) which mediate non-image-forming (NIF) effects of light such as the pupillary light reflex and complex regulations of the circadian system, sleep and [1–4]. The signal of light, the most powerful zeitgeber in human chronobiology, is projected from the ipRGCs via the retinohypothalamic tract to the suprachiasmatic nucleus which synchronizes the endogenous rhythm with an intrinsic period length averaging 24.2 hours to the exact 24-hour light-dark cycle [5–6]. In addition to this important chronobiological effect, light possesses regulatory effects on mood [2–3]. It remains unclear how closely the effects on mood and circadian rhythm are associated, or if they are indeed separate systems. Recent animal studies suggest that the effect of light on mood runs through a distinct pathway from the ipRGCs to the perihabenular nucleus and that alteration of mood can take place without alterations of sleep or circadian rhythm [2–3]. This is corroborated by the results of a randomized cross-over study including patients with depression reporting significant improvement after a single administration of bright light [7].

Glaucomatous retinal neurodegeneration has been associated with increased risk of both depression and sleep disturbances [8–11]. These associations may be attributable to a disrupted central light input as well as to the psychological burden of a chronic progressive disease and the threat of blindness. Indeed, there is evidence of attenuated NIF-functions in glaucoma such as reduced pupillary responses to blue light [12–16], reduced light induced suppression of pineal melatonin release [17] and a reduced alerting response to light [16]. These dysfunctions can all be linked to the loss of retinal ganglion cells, specifically the ipRGCs. The attenuations in the NIF-responses to light are most prominent in advanced stages of glaucoma [8,15,17] with substantial degeneration of ipRGCs and severely compromised visual field [18]. Most of the evidence for attenuations in the NIF physiologic responses to light is based on studies in primary open angle glaucoma [12,14,15,17]. The majority of studies assessing sleep and mood alterations were however performed in heterogenous populations of patients with both primary and secondary glaucomatous disease [8,9,11,19,20]. There is a lack of knowledge of how NIF functions are affected in different subtypes of glaucoma. Moreover there are no reports on the prevalence of seasonal affective disorder (SAD) in persons with glaucoma although this mood disorder is most closely associated with changes in light.

In populations living north of the equator, seasonal variation in mood and behavior is common [21–23]. Seasonality varies across a spectrum starting with a slight wintertime energy drop and moving across subsyndromal seasonal affective disorder (sSAD) with significant loss of energy, increased sleep duration and minor reductions in everyday functioning [24,25]. At the end of the spectrum is the clinically distinct syndrome of seasonal affective disorder (SAD) with recurrent major depressive episodes that cause marked reductions in daily functioning and quality of life during winter [26–28]. The depressive episode in SAD is often characterized by atypical symptoms such as hypersomnia and hyperphagia with a distinct craving for carbohydrates [26,27]. The syndrome is most prevalent among younger females [24]. In central European prevalence studies, 2–3 percent of the general populations fulfill the criteria for SAD [22,29]. Screening studies that assess self-reported seasonality using the Seasonal Pattern Assessment Questionnaire (SPAQ) tend to report higher prevalence rates compared with studies that perform clinical assessment [21,23,24]. The yearly onset of SAD symptoms coincides with reductions in the level of ambient light [30], and the disorder is effectively treated with light therapy [31]. Interestingly, persons with SAD have altered retinal light sensitivity including attenuations of the ipRGC-mediated pupillary response to blue light [32–34]. In persons with severe visual impairment or blindness, self-reported seasonality is increased in comparison with fully sighted controls [21]. No studies have assessed seasonal variation in mood and behavior in persons with glaucoma. It is plausible that a compromised light input to the central neurocircuitry caused by ipRGC degeneration can lead to increases in depressive symptoms and sleep disturbances in seasons with low levels of daylight. We therefore investigated seasonal variation in mood and behavior among outpatients with glaucoma. We examined the correlations between mood and behavior seasonality and a structural and functional measure of the degree of retinal ganglion cell damage. We hypothesized that increasing retinal ganglion cell damage would be associated with increased seasonality.

## Methods

### Study design and setting

The questionnaire study was performed among outpatients attending the glaucoma clinic at the Department of Ophthalmology at Rigshospitalet. The glaucoma clinic is a highly specialized unit receiving patients with glaucoma from the Capital Region of Denmark. On randomly selected days during the winter months of 2017 and 2018, the attending physician invited outpatients older than 18 years with a diagnosis of glaucoma to participate in the study. Eligible participants were asked to complete the SPAQ and to give their consent for the investigator to access their medical chart. A diagnosis of glaucoma was confirmed, if the clinical chart described glaucomatous cupping of the optic nerve head and corresponding visual field defects larger than 4dB. All included participants were treated with intraocular pressure (IOP)-lowering medication or had been through filtration surgery. Participants were excluded if the chart review did not reveal a diagnosis of glaucoma, if the respondent reported most discomfort during summer months, or if a consent for chart access was not obtained.

### Instrument

The SPAQ instrument rates self-reported seasonal variation (from no variation to extremely marked variation) within the 6 items: sleep duration, mood, appetite, body weight, social activity and energy level [20]. The scores (0–4 points, 4 is extreme variation) of each item are combined to a global seasonality score (GSS) ranging from 0–24. The respondent states which months are associated with most discomfort and to which degree the seasonal variation constitutes a problem for him/her. The original Kasper criteria for SAD are a GSS ≥ 11 and the symptoms should be perceived as at least a moderate problem and be present during winter months (November–February) [24]. sSAD represents a subclinical condition that cause more subtle reductions in everyday functioning than the full syndrome [25]. The Kasper criteria for sSAD are either a GSS ≥ 11 and the problem being rated as no worse than mild or a GSS of 9–10 and the problem being “mild” or worse. The Kasper criteria for both SAD and sSAD were used in the study.

### Chart data

For all respondents, we recorded data from the most recent ophthalmologic examination performed in the glaucoma clinic. The recorded data were: best corrected visual acuity (Snellen chart), IOP, lens status, and optic nerve head evaluation (cup-to-disc ratio). As a measure of disease severity, we used data from monocular visual field examination performed by Octopus perimetry where the patient reports the detection of light flashes in the central 30-degree visual field while focusing the gaze on a central fixation point. Visual field defects are reported as mean defects (MD) which are used as a clinical staging parameter of glaucomatous damage relating to retinal ganglion cell dysfunction. We considered the perimetric examination valid if the reliability factor was below 15%. If the perimetry was invalid, but recent examinations with similar results were valid, we accepted the current results. The better eye was defined as the eye with the smallest visual field defect and was used for primary analysis. As an additional measure accounting for all light input to the brain, we averaged the mean defects for both eyes. In cases where no mean defect was reported for the worse eye and visual acuity was reported as 0 or worse, we applied a value of 30 to indicate a maximally compromised visual field. We did not have restrictions on the reliability factor for the average mean defect including the autoperimetry of the worse eye. As a structural measure of the retinal ganglion cells, we applied the thickness of the peripapillary retinal nerve fiber layer (RNFLT) identified with spectral-domain optical coherence tomography (SD-OCT; Spectralis software, version 5.3, Heidelberg Engineering, Heidelberg, Germany). SD-OCT is a non-invasive scanning technique producing high resolution images of the retinal structures by use of long-wavelength light scattering.

We recorded the type of glaucoma according to four categories: primary open-angle glaucoma, primary angle-closure glaucoma, secondary glaucoma, and juvenile or congenital glaucoma.

### Ethics statement

The study was approved by the Committee on Health Research Ethics of the Capital region of Denmark (H-17027752) and by the Danish Data Protection Agency, Capital Region of Denmark (2012-58-0004) and was conducted according to the principles expressed in the Declaration of Helsinki. All participants provided written informed consent.

### Statistics

Data were analyzed in SPSS version 25. Background variables are presented as means and standard deviations. Overall correlations were tested with a Spearman correlation test. We compared seasonality scores across subgroups by nonparametric analysis due to a non-normal distribution (Mann-Whitney, Kruskal-Wallis). The age-, sex- and diagnosis-adjusted effects of the retinal measures (visual field MD and RNFLT) were tested in linear regression models, constructed with GSS as the dependent variable, gender and diagnosis as fixed factors and age and one of the retinal ganglion cell measures (visual field MD or RNFLT) as covariates. Initially, we tested the two-way interactions including the retinal measures (retinal measure x sex, retinal measure x diagnosis) and removed them if they were insignificant by removal of the variable with lowest significance first. The final model included the main effects of age, sex and diagnosis and the interaction between visual field MD and diagnosis. We applied similar models to a subgroup of persons younger than 67 years. The final model included age, sex, diagnosis and the interaction between sex and visual field MD/RNFLT. We performed visual inspection of residual plots to evaluate model fit.

An a priori power calculation revealed that 86 participants were needed to detect a simple correlation between visual field defects and seasonality score of 0.3 with a power of 0.8 and alfa level of 0.05.

## Results

A total of 137 outpatients from the glaucoma clinic completed the SPAQ. Of these, 24 persons were excluded, because 16 persons did not fulfil the inclusion criteria for glaucoma after chart review, 4 persons did not provide consent for chart access, and 4 persons described summer discomfort due to allergies/photophobia. Thus, 113 persons were included in the analysis. Median age was 66 years (IQR = 59–73) and 65 participants (58%) were women. The majority of the participants were diagnosed with primary open angle glaucoma (n = 64, 57%). Table 1 summarizes background characteristics of the participants.

**Table 1:** Clinical characteristics of the study population.

Participants, n			113		
Age, years (IQR)			66 (59 to 73)		
Female sex, n (%)			65 (58%)		
Diagnosis, n (%)					
Primary open angle glaucoma			64 (57%)		
Secondary glaucoma			30 (26%)		
Juvenile/congenital conditions			7 (6%)		
Primary angle closure glaucoma			8 (7%)		
	**Better eye**	**Missing data (n)**		**Worse eye**	**Missing data (n)**
Visual field MD (dB; mean (SD))	12.8 (6.8)	0		17,9 (6,1)	7
RNFL thickness (μm; mean (SD))	62,6 (19,1)	16		51,3 (14,6)	25
Visual acuity (mean (SD))	0,8 (0,3)	11		0,6 (0,4)	11
Cup-to-disc ratio (mean (SD))	0,7 (0,2)	29		0,8 (0,1)	36
IOP (mmHg; mean (SD))	15,1 (5,1)	15		16,2 (7,0)	18
Cataract, n (%)	18 (16%)	14		20 (18%)	17
IOL, n (%)	50 (44%)	14		49 (43%)	17

IQR = interquartile range; MD = Octopus visual field mean defect; RNFL = retinal nerve fiber layer; IOP = intra ocular pressure; IOL = intraocular lens.

**Fig 1:**
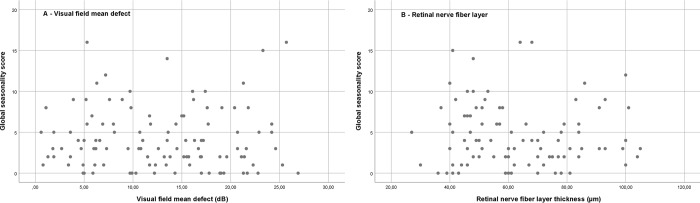
Global seasonality score and retinal ganglion cell measures. Global seasonal score plotted against visual field mean defect (A) and retinal nerve fiber layer thickness (B) of the better eye in 113 persons with glaucoma.

In the total population mean GSS was 4,3 (median 3, IQR 1.5–6.0), (see Table 2). A total of 4% (n = 5) fulfilled the Kasper criteria for SAD, and another 8% (n = 9) fulfilled criteria for sSAD. There were no differences in seasonality scores across sexes (p = 0.79) or types of glaucoma (p = 0.10). GSS was negatively correlated with age (β = -0.31, p = 0.001). There were no significant correlations between GSS and visual field MD or RNFLT of the better eye (p = 0.65, and p = 0.89, respectively) or between GSS and the average visual field MD (p = 0.33) or average RNFLT (p = 0.49) of both eyes (see Fig 1 for scatters). In the regression model adjusted for sex, age and diagnosis, the interaction between diagnosis and better eye visual field MD was significant (p = 0.02, see Table 3 for estimates). This trend was not significant when we used the average visual field MD (p = 0.06). There was no significant effect of better eye or average RFNLT on the GSS in the crude or adjusted model. The correlations between visual field MD and RNFLT were 0.62 (p<0.001) for the better eye and 0.54 (p<0.001) for the average measures of both eyes.

**Table 2:** Seasonality scores and prevalence of seasonal affective disorder in the study population (n = 113) and the subgroup under 67 years (n = 59).

	All glaucoma	Glaucoma age<67 years age <67
Participants, n	113	59
GSS median (IQR)	3 (1.5–6)	4 (3–7)
GSS mean (SD)	4.2 (3.7)	5.2 (4.0)
SAD, n (%)	5 (4.4%)	5 (8.5%)
sSAD, n (%)	9 (8.0%)	5 (8.5%)

GSS = Global Seasonal Score; IQR = interquartile range; SAD = seasonal affective disorder; sSAD = subsyndromal seasonal affective disorder.

**Table 3:** Effects of clinical characteristics on global seasonality score in all respondents (n = 113) and respondents under 67 years (n = 59).

	All glaucoma, n = 113			Glaucoma < 67 years, n = 59		
	B	95% CI	p	B	95% CI	p
**Sex**						
Male	0.71	-0.80;16.53	0.339	4.82	0.74;8.91	**0.022**
Female	0			0		
**Diagnosis**						
Primary open angle glaucoma	0			0		
Secondary	4.78	1.63;7.94	**0.003**	0.94	-1.53;3.40	0.449
Juvenile/congenital conditions	-0.50	-5.76;9.55	0.852	-1.86	-6.06;2.34	0.378
Primary angle closure glaucoma	2.05	-5.44;9.55	0.588	1.47	-2.41;5.35	0.451
**Age**	-0.09	-0.15;-0.03	**0.004**	-0.02	-0.12;-0.10	0.788
Interactions with visual field mean defect (MD*sex, MD*diagnosis)						
Male	-	-	-	-0.15	-0.37;0.08	0.191
Female	-	-	-	0.22	0.04;0.40	**0.015**
Primary open angle glaucoma	0.15	0.01;0.28	**0.035**	-	-	**-**
Secondary glaucoma	-0.24	-0.44;-0.41	**0.019**	-	-	**-**
Juvenile/congenital conditions	0.04	-0.28;0.36	0.807	-	-	**-**
Primary angle closure glaucoma	0.11	-0.41;0.62	0.675	-	-	**-**

In the subgroup aged 66 or younger (n = 59), median age was 59 years (IQR = 50–62) and 36 participants (61%) were female. For the better eye, mean visual field MD was 11,9 (SD = 7.7), mean RNFLT was 64.8 μm (SD = 20.9), mean cup-to-disc ratio = 0.77 (SD = 0.15), mean IOP = 14.6 mmHg (SD = 4.4) and mean visual acuity was 0.70 (SD = 0.34). There was sign of cataract in 13 persons (22%), and 15 persons (25%) had an intraocular lens. Regarding type of glaucoma, 26 participants (45%) were diagnosed with primary open angle glaucoma, 21 (36%) with secondary glaucoma, 5 (9%) with juvenile/congenital conditions and 7 (10%) with primary angle closure.

**Fig 2:**
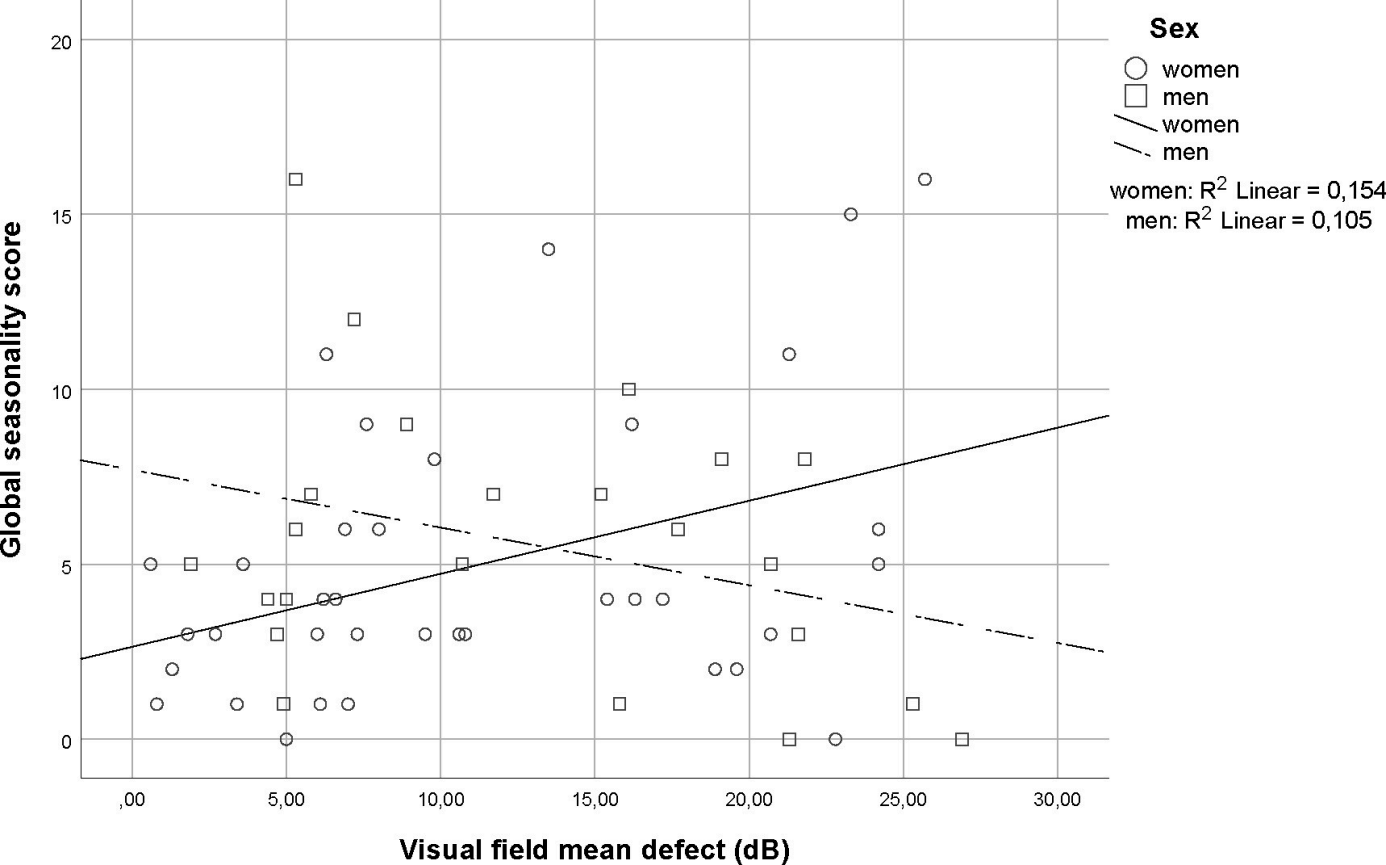
Global seasonality score plotted against perimetric mean defect for men and women ≤ 66 years. Global seasonal score plotted against visual field mean defect of the better eye of 36 women and 23 men aged 18–66 with a diagnosis of glaucoma.

There were no correlations between GSS and the better eye measures of retinal ganglion cells (visual field MD: r = 0.13 p = 0.32 and RNFLT: r = -0.12 p = 0.41). In the regression model adjusted for age, sex and diagnosis, the interaction between sex and visual field MD was significant for the better eye (p = 0.011) and for the average MD of both eyes (p = 0.028), (see Fig 2 for scatter and Table 3 for estimates). For the structural measures, the interactions between sex and RNFLT were not significant (better eye RNFLT, p = 0.070, average RNFLT = 0.439).

## Discussion

In a population of 113 outpatients with glaucoma of differing severity and etiology, we found no evidence of an association between increasing retinal ganglion cell damage and mood and behavior seasonality. There were no correlations between seasonality and a structural or functional measure of retinal ganglion cell loss. Seasonality outcomes from the SPAQ were a mean GSS of 4.3 and a prevalence of SAD and sSAD of 4% and 8%,respectively, which are rather low compared with similar screening studies of healthy populations [21–23].

Self-reported mood and behavior seasonality is known to decline with age [23–24] and the low seasonality found in our study is likely attributable to the high average age of the study population. To the best of our knowledge, no studies have investigated mood seasonality with the SPAQ in a similarly aged population. We therefore conducted a subgroup analysis of persons younger than 67 years. In this subgroup, self-reported seasonality was higher with a mean GSS of 5.2 and a prevalence of both SAD and sSAD of 8.5%. These results are only slightly higher compared with our prior findings in a Danish population (n = 2271) without known eye disorders (GSS = 4.1, prevalence of SAD = 7.6% and sSAD = 5.7) [21]. This suggests that glaucoma is not a strong risk factor for SAD. In the younger subgroup, we found that visual field defects were associated with higher GSS in women, but not in men. This trend is based on a small subsample and may constitute a type I error. The susceptibility to seasonal mood swings is, however, most prominent in younger women [23,24]. Therefore a minor effect of a compromised retinal ganglion cell function on the GSS may appear only in this subgroup.

Several studies have linked glaucoma and disturbances in mood and sleep [8–12,20,35–39]. Visual field defects are associated with lower sleep quality, increased daytime-sleepiness and higher levels of self-reported anxiety and depression and [8–10, 35]. The reported associations are rather weak which may in part be due to inclusion of patients with minor visual field defects and different type of glaucoma. In our study, we found a trend towards a differential association between seasonality scores and visual field defects in different types of glaucoma. If this finding is corroborated, sleep and non-seasonal mood regulation may also be differentially compromised in different types of glaucoma. A substantial loss of RGCs is required before NIF functions are compromised, and the ipRGCs are resistant to degeneration compared with conventional retinal ganglion cells [40]. Indeed, the density of melanopsin-staining ipRGCs is reduced in enucleated eyes with severe glaucomatous damage but unaffected in eyes with early glaucoma [18]. The density of ipRGCs is, however, reduced in both primary and secondary glaucoma. Correspondingly, the NIF effects of light, specifically the sustained pupillary response to short wavelength light and the light induced suppression of pineal melatonin, are most severely affected in advanced stages of glaucoma [15–17]. In our population, approximately 55% had visual field defects worse than 12 dB in the better eye indicating an advanced stage of disease. It may be that the remaining respondents with earlier stage disease maintain sufficient ipRGC-function why we do not see an overall increase in seasonality. Other studies have also failed to detect alterations of mood ad behavior outcomes in glaucoma. Chronotype did not differ between persons with glaucoma and healthy controls, although the variability in chronotype tended to increase with glaucoma severity [41]. Again, the sample included both primary and secondary glaucoma and the results were not adjusted for type of glaucoma. A large population- based study assessed depressive symptoms in persons with or without self-reported glaucoma and found no differences between the two groups [19]. Again, the study did not assess the severity or subtype of glaucoma and was performed in a population-based sample why both persons with severe depression and advanced glaucoma may have evaded participation.

The present study presents some limitations. First and foremost, the survey was performed in a cross-sectional design in a small heterogenous sample. Secondly, the results are based on a self-reported retrospective measure prone to some recall bias. The study setting in Northern Europe limits the relevance to locations with less pronounced seasonal variation in photoperiod and light exposure. Seasonal variation in mood and behavior is increased in both seasonal and non-seasonal depression [42]. The present survey contains no assessment of non-seasonal depression although this condition is prevalent in glaucoma [11]. We cannot establish whether a lowering of mood and energy during the dark seasons is related to an insufficient central light input or to limitations in mobility and physical activity caused by poor lighting. Glaucomatous visual field defects are primarily peripheral and therefore constitute a challenge to orientation sight. In combination with poor lighting, visual field defects could cause reductions in daily functioning and quality of life that may in turn have depressogenic effects. We would however not expect such an effect to present only in subgroups.

In conclusion, mood and behavior seasonality was not significantly associated with decreasing visual field or retinal ganglion cell damage. Since glaucoma affects the neurobiological system beyond the visual function, further insight into the association between retinal function, mood and sleep may help ensure proper patient care. We found trends for differential effects of retinal ganglion cell dysfunction on seasonality in subgroups with different sex and type of glaucoma. Further studies are needed to confirm whether specific subgroups are more prone to NIF dysfunction and consequently could have an increased vulnerability to mood and sleep disorders.

## Supporting information

S1 File Data underlying the study. (XLSX)
